# Effect of Ureteral Stent Length and Position of Stent Coil in Bladder on Stent-Related Symptoms and Quality of Life of Patients

**DOI:** 10.7759/cureus.11669

**Published:** 2020-11-24

**Authors:** Ketan Mehra, Ramanitharan Manikandan, Lalgudi N Dorairajan, Sreerag Sreenivasan Kodakkattil, Sidhartha Kalra

**Affiliations:** 1 Urology and Renal Transplantation, Jawaharlal Institute of Postgraduate Medical Education and Research, Pondicherry, IND; 2 Urology and Renal Transplantation, Jawaharlal Institute of Post Graduate Medical Education and Research, Pondicherry, IND

**Keywords:** ureteral stent symptom, stent, ureter, calculus

## Abstract

Introduction: Various standardized questionnaires can evaluate ureteral stent-related symptoms. The present study utilized a validated instrument, Ureteral Stent Symptoms Questionnaire (USSQ), to know the impact of the length of double J stent (DJS) in comparison to ureter length on patients and their quality of living.

Materials and Method: This study is a prospective clinical study conducted in the Department of Urology at a tertiary care center in South India. Patients who underwent DJS after endoscopic ureteral lithotripsy were included in the study. On a computerized tomography scan, the ureteral to stent length ratio (USR) was calculated. USSQ scores at the time of DJS removal and two weeks thereafter were recorded. The distal coil of the stent in the bladder was recorded as grade 1 - not crossing the midline and grade 2 - crossing the midline. Different symptom scores were compared between both grades of bladder coil and for USR of all the patients.

Result: A total of 157 patients were included in the study. Over 46 (29.3%) patients had grade-1 and 111 (70.7%) had grade-2 bladder coil. Totally 93 (59.23%) patients reported pain, while 64 (40.77%) patients had no pain. Grade-2 coil patients had more pain than grade 1 (P=0.01). There was a weak inverse relationship between the USR and urinary symptom (P=0.004), pain symptom (P=0.04), and quality of work (P=0.005).

Conclusion: Stent length or position of the intravesical stent coil does not appear to affect the quality of life except for the pain. Hence, choosing stent length according to ureteral length seems to have a minimal role in decreasing stent-related morbidity.

## Introduction

The endoluminal stent was first described by Zimskind et al. [1]. They used silicone rubber tubing for treating patients with carcinomatous obstruction of the ureter, uretero-vaginal fistula, and ureteral stricture. Patients undergoing ureteral double J stenting (DJS) for various pathologies experience symptoms like pain, lower urinary tract symptoms (LUTS), sexual dysfunction, and altered work capacity attributed to the DJS in situ. More than 80% of patients suffer from stent-related pain, which interferes with daily activities. Furthermore, 32% of these patients also experience sexual dysfunction, and 58% reported reduced work capacity with a negative economic impact [2]. One of the main determinants reported in the literature for developing such symptoms is a longer stent length with an excessive coil of the lower part of the urinary bladder's stent. Hence, choosing a stent of an appropriate length to match the patient's ureteric length becomes essential [3]. The vast majority of patients who require DJS present with a preoperative computerized tomography (CT) scan. Hence, a CT scan can be a useful tool to measure the ureteral length so that the appropriate length of the stent to be deployed can be determined preoperatively [4]. The symptom assessment after DJS can be performed using a validated instrument, the Ureteral Stent Symptoms Questionnaire (USSQ), that evaluates symptoms and quality of life affection [2]. It consists of 38 items in six domains. The aim of the present study is to evaluate the effects of stent length and position of the bladder coil on the patient's symptoms by using a validated questionnaire.

## Materials and methods

The present study is a prospective observational study conducted in the Department of Urology at a tertiary health center in South India. The study period ranged from January 2017 till December 2018 for two years. All eligible patients were informed about the opportunity to be recruited into the study with confidentiality maintained. Institutional Ethics Committee approval was taken. The patients between the ages of 18 and 70 years were included in the study. Patients with urinary tract infection (UTI), benign prostatic hyperplasia (BPH), pregnancy, ureteral anomaly, previous ureteral surgery, painful symptoms due to other cause, or inflammatory diseases like a viral infection, hepatitis, gastroenteritis, active neoplasia, cardiovascular disease, hypertension, obesity, diabetes, liver failure, and bilateral DJS were excluded.

All patients in the study underwent ureteroscopy by 9 French semirigid ureteroscope and pneumatic lithotripsy under proper anaesthesia. The DJS was performed at the end of the procedure. Three various sizes of DJS, 22 cm, 24 cm, and 26cm, were used for stenting in all patients. All stents used were of 5 French and of the same manufacturing company. The ureteral length was measured on non-contrast computerized tomography (NCCT) scan from the renal vein to the vesicoureteric junction (VUJ). The ratio of the ureter to stent length was then calculated. The stent was removed after two weeks of surgery. At the time of DJ stent removal, the patients were asked to complete the Ureteral Stent Symptom Questionnaire (USSQ). At the same time, the position of the bladder coil of the stent was noted fluoroscopically before removing the stent and graded as grade 1 - distal coil not crossing the midline of the bladder. Grade 2 - distal coil crossing the midline of the bladder. The ratio of ureteral to DJS length and grade of stent coil in the bladder was used to compare USSQ outcomes and other grouping variables like age and gender. The patients were called for follow-up after two weeks of stent removal and again requested to complete the USSQ to use as a control.

Data collection was performed on a continuous basis. The distribution of data on categorical variables such as gender, the grade of stent coil in the bladder, symptoms like whether pain was present or not, global quality of life, and additional problems was expressed as frequencies and percentages. The continuous data such as age, ureteral to stent length ratio (USR), USSQ score like Pain Index Score (PIS), Urinary Index Score (UIS), General Health Index Score (GHIS), Sexual Symptom Score, and days of work lost were expressed as mean with standard deviation (SD) or median with range.

The comparison of the USR, the position of the coil in the bladder, and USSQ scores was carried out by using an independent t-test/Mann-Whiney U- test. The relationship of the USR, the position of the coil in the bladder, and the USSQ score were explored using correlation analysis. The statistical significance of the difference in the USSQ score over time was carried out using a paired t-test or Wilcoxon's signed rank-sum test. All statistical analyses were carried out at a 5% level of significance, and a p-value < 0.05 was considered significant. The sample size was estimated using the statistical formula for assessing the linear relationship of USR, stent coil in the bladder, and USSQ score as 0.65 with an expected population correlation coefficient of 0.50 with 80% power and 5% level of significance. Statistical analysis was performed using IBM SPSS Statistics 48 for Windows, version 19 (IBM Corp., Armonk, NY).

## Results

A total of 157 patients were eligible to be included in the study. The mean age was 42.82 years ± 13.93 years. There were 95 males (60.5%) and 62 females (39.5%). The ureteral length measured on CT scan ranged from 148 mm to 293 mm with a mean of 202.75 ± 26.38 mm. In males, the mean ureteral length was 203.6 ± 26.4 mm, and in females, it was 201.44 ± 26.5 mm. The USR ranged from 5.69 to 11.27, with a mean of 7.79 ± 1.01 (SD). In males, the mean ratio was 7.83 ± 1.01, and in females, 7.74 ± 1.0. 46 (29.3%). Other characteristics like the body metabolic index, the presence or absence of proximal hydronephrosis, the position of stone proximal or distal to iliac vessels crossing were recorded (Table 1). Both the groups were comparable in the above attributes.

**Table 1:** Demographic description SD: standard deviation.

Demographic parameters	Central tendency
Mean age in years ± SD	42.82 ± 13.93
Mean age in males in years ± SD	44.06 ± 13.90
Mean age in females in years ± SD	40.92 ± 13.87
Number of males (%)	95 (60.5)
Number of females (%)	62 (39.5)
Mean ureteral length in mm ± SD	202.75 ± 26.38
Mean ureteral stent length ratio ± SD	7.79 ± 1.01
No. of patients with stent coil not crossing the midline of the bladder (grade 1)	46 (29.3%)
No. of patients with stent coil crossing midline of the bladder (grade 2)	111 (70.7%)
Median body metabolic index (range)	20 (16-30)
Median size of stone in the ureter in cm (range)	1.1 (0.7-2.4)
Median size of stone in grade 1 coil group in cm (range)	1.05 (0.7-2.4)
Median size of stone in grade 2 coil group in cm (range)	1.1 (0.7-2.1)
No. of cases with hydronephrosis proximal to stone (%)	6 (3.8)
No. of cases with proximal calculus (%)	86 (54.8)
No. of cases with distal calculus (%)	71 (45.2)

Over 93 (59.23%) patients reported pain, while 64 (40.77%) patients had no pain. 20 (43.5%) patients with grade 1 coil, and 73 (65.8%) patients with grade 2 bladder coil reported pain (P=0.01). Concerning gender, the association of grades of stent coil and pain was significant in males (Table 2).

**Table 2:** Association of stent coil in bladder and pain

Patient group	Outcome variable	Grade 1 (not crossing the midline)	Grade 2 (crossing the midline)	P-value
In all patients (n=157)	Did not have pain	26 (56.5%)	38 (34.2%)	0.01
	Had pain	20 (43.5%)	73 (65.8%)	
Males (n=95)	Did not have pain	16 (61.54%)	22 (31.89%)	0.009
	Had pain	10 (38.46%)	47 (68.11%)	
Females (n=42)	Did not have pain	10 (50%)	16 (27.78%)	0.375
	Had pain	10 (50%)	26 (72.22%)	

On the application of the PIS in the above subset of patients, there was no difference in the mean PIS score between grades 1 and 2 stent coil (Table 3). Similarly, there was no difference in the values of UIS, GHIS, median days or half days lost, score for quality of work, and Sexual Symptom Score between the two groups of bladder coil (Table 3).

**Table 3:** Association of the position of stent coil in bladder with different outcome scores obtained from USSQ USSQ: Ureteral Stent Symptom Questionnaire, PIS: Pain Index Score, UIS: Urinary Index Score, GHIS: General Health Index Score.

Outcome variable	Grade 1 (not crossing the midline)	Grade 2 (crossing the midline)	P-value
Mean PIS ± SD	22.45 ± 3.85 (n=20)	22.92 ± 4.23 (n=73)	0.319.
Mean UIS ± SD	22.87 ± 2.83 (n=46)	22.87 ± 2.83 (n=111)	0.078
Median GHIS (range)	12 (6-21) (n=46)	12 (6-26) (n=111)	0.435
Median days lost (range)	1 (0-3)	1 (0-6)	0.080
Median half days cut (range)	0 (0-1)	0 (0-1)	0.152
Median score for quality of work (range)	7 (5-9) (n=44)	7 (5-12) (n=99)	0.757
Median Sexual Symptom Score	4 (3-11) (n=39)	4 (2-11) (n=82)	0.877

Under the GQoL section, none of the patients reported “delight” with the stent in situ. The majority of the patients in both groups had either “mixed feelings” or were “mostly satisfied.” Nine (19.5%) patients in grade 1 and 27(24.3%) in grade 2 group felt either “dissatisfied,” “unhappy,” or “terrible” (P=0.534).

Under the additional problem questionnaire section, 35.1% of patients reported symptoms suggestive of infection at some point in time, and this was not found to be different among the stent coil groups. Among grade 1, 41.3% of patients had to take antibiotics for infection compared to 44.1% in grade 2 (P=0.013). There was no significant difference between both the groups when asked about how many times they had to visit the healthcare professional or had to get admitted due to the stent-related symptoms.

The ureteral length was measured on a CT scan, and the USR was calculated and analysed for both grades 1 and 2 of the stent coil in the bladder. In patients with grade 1 stent coil, the USR was 8.32 ± 1.07(SD), while in the grade 2 group, the ratio was 7.58 ± 0.9, which was statistically significant (P<0.001). The association between pain or no pain and ureter to stent length ratio (USR) was analysed. The mean USR in patients who had pain was 7.64 ± 0.96, and in those who felt no pain, the mean USR was 8.01 ± 1.05 (P=0.024).

Coefficient results indicate a weak inverse relationship with significant difference between the USR and UIS (r=-0.230, P=0.004, n=157), PIS (r=-0.164, P=0.04, n=93) and quality of work (r=-0.232, P=0.005, n=143). While half days cut down had an inverse relation with USR, but the difference was not significant (r=-0.107, P=0.184, n=157). General health Index Score (GHIS) had a weak positive linear relationship with USR and had a significant difference (r=.220, P=0.006, n=157). Other outcome variables like days in bed (r=.025, P=0.761, n=157) and quality of sex (r=.168, P=0.066, n=121) had a weak positive linear relationship, but the difference was not significant.

In the subsection of GQoL, 77% of all the patients with DJ stent in situ were satisfied, the rest 33% had some level of dissatisfaction. The association between different categories of the GQoL like “pleased to terrible” and median USR was not significant (P=0.555). The association between different sections of additional problems and USR suggested that the number of times a patient had to take antibiotics had a significant association, with 43.5% of patients had to take antibiotics (P=0.003).

The PIS and UIS had a significant difference in patients with the stent in situ and without stent (P<0.001; Table 4).

**Table 4:** Comparison of pain and urinary scores on follow-up PIS: Pain Index Score, UIS: Urinary Index Score.

Score compared	Scores with stent/ without stent	Median (range)	P-value
PIS	PIS with a stent in situ	18 (0-35)	<0.001
	PIS post stent removal	0 (0-25)	
UIS	UIS with a stent in situ	23 (16-37)	<0.001
	UIS post stent removal	15 (12-30)	

The association of stent coil grades in the bladder and individual scoring items in UIS, PIS, and GHIS was carried out (Table 5). 

**Table 5:** Association of urinary index individual score with a grade of stent coil in the bladder

Category (question number in the questionnaire)	Grade of bladder coil	Median score (range)	P-value
Frequency (U1)	Grade 1	1	O.472
	Grade 2	1	
	Total	1	
Nocturia (U2)	Grade 1	2	0.088
	Grade 2	3	
	Total	3	
Urgency (U3)	Grade 1	3	0.329*
	Grade 2	2	
	Total	3	
Urge Incontinence (U4)	Grade 1	2	0.564
	Grade 2	2	
	Total	2	
Incomplete evacuation of bladder (U6)	Grade 1	2	0.945
	Grade 2	2	
	Total	2	
Dysuria (U7)	Grade 1	2	0.523
	Grade 2	2	
	Total	2	
Incidence of haematuria (U8)	Grade 1	1	0.620
	Grade 2	1	
	Total	1	
Extent of haematuria (U9)	Grade 1	1	0.675
	Grade 2	1	
	Total	1	
Overall urinary symptoms problem (U10)	Grade 1	2	0.014
	Grade 2	2	
	Total	2	
Pain interrupting sleep (P5)	Grade 1	3	0.837
	Grade 2	2	
	Total	2	
Pain while passing urine (P6)	Grade 1	2	0.797
	Grade 2	3	
	Total	3	
Pain in kidney while passing urine (P7)	Grade 1	1	0.638
	Grade 2	2	
	Total	2	
Painkillers for controlling pain (P8)	Grade 1	2	0.501
	Grade 2	2	
	Total	2	
How much pain interferes with life (P9)	Grade 1	3	0.492
	Grade 2	3	
	Total	3	
Difficulty in performing light physical activity (G1)	Grade 1	1	0.314
	Grade 2	1	
	Total	1	
Difficulty in performing heavy physical activity (G2)	Grade 1	1	0.120
	Grade 2	2	
	Total	2	

Among all individual scores, only overall urinary symptoms causing problems significantly differed among the grades. Opposite to expectation, grade 1 group 30.4% of patients had “moderate,” “quite a bit” or “extreme” problem compared to grade 2 in which only 18.9% had such type of urinary problems (Table 6).

**Table 6:** Overall urinary symptoms as a problem in grades 1 and 2 of stent coil in the bladder

Category	Grade 1 (not crossing midline)	Grade 2 (crossing the midline)	Total	P-value
Not at all	1 (2.2%)	21 (18.9%)	22 (14%)	0.014
A little bit	31 (67.4%)	69 (62.2%)	100 (63.7%)	
Moderate	11 (23.9%)	13 (11.7%)	24 (15.3%)	
Quite a bit	3 (6.5%)	7 (6.3%)	10 (6.4%)	
Extreme	0 (0%)	1 (0.9%)	1 (0.6%)	
Total	46	111	157	

Lastly, the association of PIS, UIS, GHIS, Quality of Work score (QoWS), and Quality of Sex score (QoSS) with confounding factors like the position of stone (proximal or distal) and the presence or absence of hydroureteronephrosis was evaluated. There was no significant difference between the position of the stone or hydronephrosis with any of the mentioned scores. There was no linear correlation between stone size and PIS (r=0.009, P=0.910), UIS (r=0.05, P=0.53), GHIS (r=-0.088, P=0.273), QoWS (r=-0.017, P=0.845), or QoSS (r=-0.030, P=0.742).

## Discussion

We examined the influence of stent coil position in the bladder and USR on the discomfort caused by double J ureteral stents using a questionnaire explicitly devised to assess stent-related symptoms (USSQ). We found that patients experienced significantly more pain when the bladder coil of the stent crossed the midline. This difference was significant in the male population. But the difference between PIS was not significant between the subgroups. Among additional problems assessment, only the number of times patients needed to take antibiotics had a considerable difference with the one-time antibiotic course and three-time antibiotic course more in those with stent coil crossing midline. On the question "Overall, how much were the urinary symptoms bothersome," the patients who had stent coil not crossing the midline seems to have more trouble as opposed to expectation. This may be due to confounding factors like the use of anticholinergic or alpha-blockers, which were not taken into consideration. Other scores related to urinary, day to day working, sexual matters, general health, or other additional problems had no significant difference between the two groups. Frequency and nocturia (reflected by questions U1 and U2) and severity of gross haematuria (U8 and U9) between grades 1 and 2 had no difference in stent coil in the bladder, which seem to argue against more significant irritation caused by longer intravesical stents.

It was evident that a shorter ureter leading to the lesser USR leads to a stent coil in the bladder crossing midline more often. This cannot be implied that longer lengths of the stent will cross the midline in the bladder as the proximal coil can also get displaced in the upper calyx. There was also a significant association between the USR and the presence of pain. The lower USR had an inverse correlation with pain, inferring that longer stents caused more incidence of pain. Similarly, there was a weak inverse correlation between USR and PIS, UIS and QoWS, which was significant. This states that shorter ureteral lengths compared to stent length may have more pain and urinary symptoms which may affect the quality of work. The General Health Index score showed a weak direct correlation with USR. This positive correlation may be due to the use of medications like alpha-blockers, antimuscarinics, or other pain killers prescribed by the community doctors or general practitioners, which could have made patients feel better. In the GQoL, most of the patients had mixed feelings with the stent, while some were “mostly satisfied,” and few of them being “unhappy or dissatisfied.” USR also had a significant association with the number of times patients had to use antibiotics with USR lesser than the median value (<7.69) had to take antibiotics frequently. The relation of coil position and USR with the use of antibiotics seems to be spurious as UTI was not proven, and it was only a patient-reported event. It is a possibility that the community physician would have wrongly interpreted stent symptoms as due to UTI and would have prescribed antibiotics. There was no significant effect of confounding factors like the stone size, the stone's position, the presence, or the absence of hydroureteronephrosis on the stent-related symptoms.

Abt et al. used the German version of USSQ in 73 patients [5]. USSQ was completed with the ureteral stent in situ the day before stent removal. Intravesical stent position was classified into three categories: the distal loop of the stent completely ipsilateral to the line, crossing the perpendicular line, or completely contralateral. The USSQ subscores for urinary symptoms, body pain, general health, work performance, and additional problems did not differ significantly between the three groups. In this study, the intravesical stent position showed no significant influence on associated morbidity. But unlike our study, Abt et al. did not assess the symptoms after stent removal, which could have allowed a better causal link between symptoms and the indwelling stent.

Giannarini et al. prospectively studied 86 patients and used the same questionnaire (USSQ) with an assessment of symptoms and stent position on days 7 and 28 after placement [6]. Body mass index (BMI), gender, and stent caliber were compared. At day 7, gender, BMI, and stent caliber were significantly associated with general health, body pain, and work performance, respectively, while on day 28, BMI was significantly associated with two domains, pain, and general health scores. On days 7 and 28, the stent coil's location in the bladder was significantly associated with urinary symptoms, body pain, general health, work performance, and sexual matters scores. The distal loop's location with respect to midline had a significant association with most domains of the USSQ on both days 7 and 28 after stent placement.

In a similar study of 59 patients by Ho et al., stents coil crossing the midline was significantly associated with urge incontinence and bladder pain [7]. They used 22, 24, and 26 cm stents in their patients. The severity of haematuria was strongly associated with 24-cm and 26-cm stents. They concluded that the length of the stent and crossing the midline of the distal end were significantly related to stent-related symptoms. The proper length of the stent is the most critical factor in minimizing stent-related symptoms.

In a multicenter study by Lingeman et al., 236 patients were distributed in four arms of short loop tail stent (60), the long loop tail stent (59), the Percuflex Plus stent (64), and the Polaris stent (53) [8]. They concluded that patients stented with the short loop tail had lower USSQ pain scores on day 4 after placement and lower pain medication use on day 1 after placement when pain peaked in all stent groups. But it was not statistically significant.

In a systematic review and metanalysis, Betschart et al. analysed 107 records and revealed treatment methods and prevention of ureteral stent-related morbidity [9]. Many of the trials using the USSQ did not provide the actual numbers for USSQ total scores and subscores. Further use of self-designed questionnaires prevented systematic comparison. The majority of trials pointed at the advantages of the shorter stent ends within the bladder, just like our study. They concluded that patients who were given more education regarding symptoms caused due to stent showed a signiﬁcant reduction of the USSQ total score and the subscore for urinary symptoms, and further well-defined trials are required to know which stent characteristics are deﬁnitely responsible for stent-related symptoms.

There were some advantages and limitations of the study. The main advantage of the study was its prospective design, and the number of patients studied. Most of the earlier studies quoted had a lesser number of patients recruited than our research. Another gain was that all the scores and subscores of the questionnaire are presented, which were not reported in the previous studies [9].

There were some limitations to our study. First, the stent coil in the bladder was assessed just once at the time of stent removal, and this was taken as the representation of the stent coil throughout. But this could be fallacious as the stent position may change with time. Second, our study included only the length and position of the stent coil in the bladder. Other factors that could contribute to stent-related symptoms like the thickness of the stent, the use of medications like alpha-blockers or antimuscarinics, and the position of the proximal coil of the stent were not considered. These factors could contribute to confounding factors affecting the results of the study.

## Conclusions

Ureteral length compared to stent length and position of intravesical stent coil can predict stent-related morbidity only in terms of pain. Proper sizes of the stent can decrease the patients' pain. The length of the stent or the position of the stent coil in the bladder does not seem to affect the general health, sexual health, work-related loss, or other urinary symptoms. According to ureteral length, matching stent length appears to have minimal or no role in ameliorating major stent-related symptoms.
